# Dynamic Yarn-Tension Detection Using Machine Vision Combined with a Tension Observer

**DOI:** 10.3390/s23083800

**Published:** 2023-04-07

**Authors:** Yue Ji, Jiedong Ma, Zhanqing Zhou, Jinyi Li, Limei Song

**Affiliations:** 1 School of Control Science and Engineering, Tiangong University, Tianjin 300387, China; 2 Tianjin Key Laboratory of Intelligent Control of Electrical Equipment, Tiangong University, Tianjin 300387, China; 3 School of Electrical Engineering, Tiangong University, Tianjin 300387, China

**Keywords:** yarn tension, machine vision, tension observer, fusion algorithm, non-contact detection

## Abstract

Machine vision can prevent additional stress on yarn caused by contact measurement, as well as the risk of hairiness and breakage. However, the speed of the machine vision system is limited by image processing, and the tension detection method based on the axially moving model does not take into account the disturbance on yarn caused by motor vibrations. Thus, an embedded system combining machine vision with a tension observer is proposed. The differential equation for the transverse dynamics of the string is established using Hamilton’s principle and then solved. A field-programmable gate array (FPGA) is used for image data acquisition, and the image processing algorithm is implemented using a multi-core digital signal processor (DSP). To obtain the yarn vibration frequency in the axially moving model, the brightest centreline grey value of the yarn image is put forward as a reference to determine the feature line. The calculated yarn tension value is then combined with the value obtained using the tension observer based on an adaptive weighted data fusion method in a programmable logic controller (PLC). The results show that the accuracy of the combined tension is improved compared with the original two non-contact methods of tension detection at a faster update rate. The system alleviates the problem of inadequate sampling rate using only machine vision methods and can be applied to future real-time control systems.

## 1. Introduction

Yarn tension during the spinning process is an important parameter that is highly correlated with yarn performance and fabric quality in production links [1]. Yarn tension during actual production is not constant, but fluctuates continuously during the spinning process. The abnormal fluctuations lead to changes in yarn properties, which have strict requirements for real-time and accurate tension detection. Through real-time tracking and control of yarn tension, the tension under each operating condition can be maintained within a constant range [2,3]. Therefore, the failures caused by aberrant tension, such as sticking and shutting down of textile machines, can be avoided, thus improving the automation level of the spinning industry and creating economic benefits [4].

Contact tension sensors are currently the typical devices used for measuring yarn tension in the textile industry. Designing sensors using surface acoustic wave (SAW) oscillators is a hot topic in the research on contact tension detection, and the main principle is that the oscillation frequency of the SAW oscillator varies with yarn tension [5,6,7]. Research on tension sensors has been conducted for many years, with good results on yarn-tension detection. However, this measurement method causes additional friction on the yarn, impacting yarn quality. In addition, in some situations, non-contact measurement is required for textile composite materials [8,9].

In many parts of the textile process, the winding of yarn is mostly carried out in an axial motion to achieve the corresponding workflow [10]. Dynamic analysis of axially moving string has received attention in many studies on the winding process in various engineering areas [11,12]. Yarn tension detection has been achieved using a laser doppler vibrometer (LDV) [13,14]. This method is novel and enables non-contact detection. Machine vision technology can determine characteristics of the tested products and is widely used in the textile industry [15,16,17]. On this basis, Wang et al. proposed an optical method for automatically measuring the microtension of yarns. The moving yarns in a winding system can be illuminated using a line laser, and a line scan industrial camera can be used to acquire images [18]. However, there are some defects which the mathematical model does not take into account the influence of other variables during motor operation. Peng et al. established a yarn-tension detection method based on transverse frequency [19]. Although non-contact detection can be achieved, the results are discontinuous and discrete. It is difficult to meet the demand for real-time tension detection in practical production. Besides, the methods only use computer software to process image data, which is not suitable for on-site control in the spinning industry. Thus, to achieve real-time detection and subsequent use in control systems, it is important to increase the tension sampling rate and complete the project using high-performance processors. Due to their strong data computing capabilities and flexible functions, digital signal processors (DSP) and field-programmable gate arrays (FPGA) have become the two most widely used processors in embedded image processing systems [20]. Herein, the tension estimated using the machine vision is implemented in a heterogeneous processing system based on the axially moving string model and image data processing methods.

In addition, PLCs are usually the main part of automatic industrial systems. They are very efficient and reliable in the manufacturing, chemical and process industries [21]. Tension observer can be realized in programmable logic controller (PLC) and has a high-tension observation rate. It has also been studied by many scholars to measure the tension of winding materials for sensorless control [22,23,24]. Hou et al. designed a decentralized coordinated control scheme with tension observers for a motor winding system. The tension observers were established to observe the winding tension [25]. Kang et al. designed a reduced dimensional observer for estimating tension over a wide frequency range [26] and the performance of the observer was analysed using only simulations. These researches have promoted the development of the tension observer model, but there are many factors that affect yarn winding tension under actual working conditions. Therefore, it will be combined with the tension estimated using the machine vision in a PLC to supplement the insufficient sampling rate of machine vision and improve the mathematical model.

Herein, a dynamic yarn-tension detection system using machine vision combined with a tension observer is made for high sampling rate and detection accuracy. The rest of this paper is organized as follows: Section 2 presents a mathematical model for the entire system and analysis. The image data processing method for machine vision tension detection is introduced in Section 3. The implementation process of tension-observer tension observation and data fusion is shown in Section 4. Section 5 contains the full experimental test platform description, while Section 6 discusses the test results. Finally, the conclusion is presented in Section 7.

## 2. Mathematical Models

### 2.1. Axially Moving String Model

**Figure 1:**
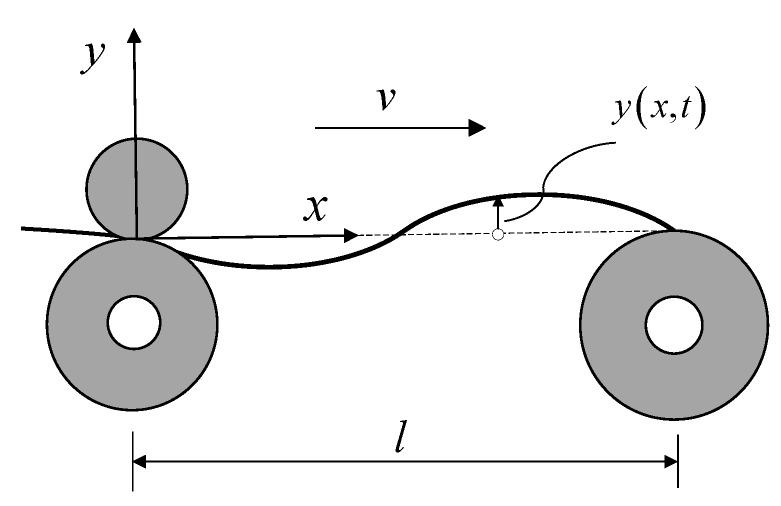
Axially moving string model.

The yarn moving between the conveying roller and the winding roller during the spanning process is equated to an axially moving string. The yarn is stretched by the rolls to produce vibrations, the frequency of which is correlated with the characteristics of the web and the tension [27]. The axially moving string model is shown in Figure 1 and enables us to obtain

(1) $$E_{k}=\frac{1}{2}\int_{0}^{l}\rho \left[{\left(vy_{x}+y_{t}\right)}^{2}+v^{2}\right]dx$$

(2) $$E_{p}=\frac{1}{2}\int_{0}^{l}Fy_{x}^{2}dx$$

where *E_k_* is the kinetic energy; *E_p_* is the potential energy; *ρ* is the yarn density; *v* is the velocity of axial motion; *l* is the length stretched along the *x*-axis; *x* represents the coordinates along the yarn direction; *F* is the tension; *y* (*x*, *t*) represents the transverse movement of the yarn at the spatial coordinate *x* at time *t*; *y_x_* represents partial derivative with respect to *x*; and *y_t_* represents partial derivative with respect to *t*.

Defining the Lagrangian $L=E_{k}-E_{p}$, Hamilton’s principle can be written as

(3) $$\delta \int_{t_{1}}^{t_{2}}Ldt=0$$

Letting $c^{2}=\frac{F}{\rho }$, the partial differential equation for the axially moving string is obtained [28], and the joint boundary conditions are written as

(4) $$\left\{\begin{array}{l} \left(c^{2}-v^{2}\right)\frac{\partial^{2}y}{\partial x^{2}}-2v\frac{\partial^{2}y}{\partial x\partial t}-\frac{\partial^{2}y}{\partial t^{2}}=0\\ y\left(0,t\right)=0,\;y\left(l,t\right)=0 \end{array}\right.$$

where *l* is the distance between the conveying roller and the winding roller. A modal solution is assumed as

(5) $$y\left(x,t\right)=R\left[W\left(x\right)e^{i\omega t}\right]$$

where *W*(*x*) is the eigenfunction, ω is the circular frequency, and *R*[·] denotes the real part. Substituting this solution in Equation (4), the eigenvalue problem for the motion string as

(6) $$-\left(c^{2}-v^{2}\right)W^{\prime \prime }+2i\omega vW^{\prime }-\omega^{2}W=0$$

The eigenfunction *W*(*x*) is complex, substituting the solution form *W*(*x*) = *Be^ikx^* in Equation (6), gives

(7) $$\begin{array}{l} \left(c^{2}-v^{2}\right)k^{2}-2\omega vk-\omega^{2}=0\\ \Rightarrow k_{1}=-\frac{\omega }{c+v}\;\mathrm{or}\;k_{2}=\frac{\omega }{c-v} \end{array}$$

Using Equation (7), the general solution can be written as

(8) $$W_{\left(x\right)}=De^{-\frac{i\omega x}{c+v}}+Ee^{\frac{i\omega x}{c-v}}$$

where *D* and *E* are arbitrary constants. Using the boundary conditions, the characteristic equation for the motion string can be written as

(9) $$e^{i\omega l\left[2c/\left(c^{2}-v^{2}\right)\right]}-1=0$$

Thus, the eigenvalues for the axially moving string is given as

(10) $$\omega =\frac{n\pi }{cl}\left(c^{2}-v^{2}\right)\;n=1,2,\ldots ,\infty$$

Using Equation (10), the relationship between string tension and the circular frequency is written as

(11) $$c=\sqrt{\frac{F_{s}}{\rho }}=\frac{l\omega +\sqrt{{\left(l\omega \right)}^{2}+4{\left(\pi v\right)}^{2}}}{2\pi }$$

Since the process of adjusting the tension of the conveying rollers affects the moving yarn, the theoretical relationship between the yarn tension, speed and vibration frequency is subsequently modified as

(12) $$F_{s}=\rho \frac{2l^{2}\omega^{2}+2l\omega \sqrt{{\left(l\omega \right)}^{2}+4{\left(\pi v\right)}^{2}}}{4\pi^{2}}+F_{f}=\rho \left(2l^{2}f^{2}+v^{2}+2lf\sqrt{l^{2}f^{2}+v^{2}}\right)+F_{f}$$

where $f=\frac{\omega }{2\pi }$ is the yarn vibration frequency; *F_s_* is the yarn tension calculated by machine vision and *F_f_* is the additional friction of the conveyor rollers. Its value is determined by fitting the functional relationship of yarn tension, vibration frequency and spinning speed. The key to calculating the yarn tension is to obtain the yarn vibration frequency.

The approximate general estimation model is obtained from the axially moving string model, and the yarn tension is calculated using the yarn’s transverse vibration frequency and the running linear velocity. Only the vibration frequency of the yarn and the speed are considered, without taking into account the disturbance formed by the speed of the motor during operation.

### 2.2. Design of the Tension Observer

**Figure 2:**
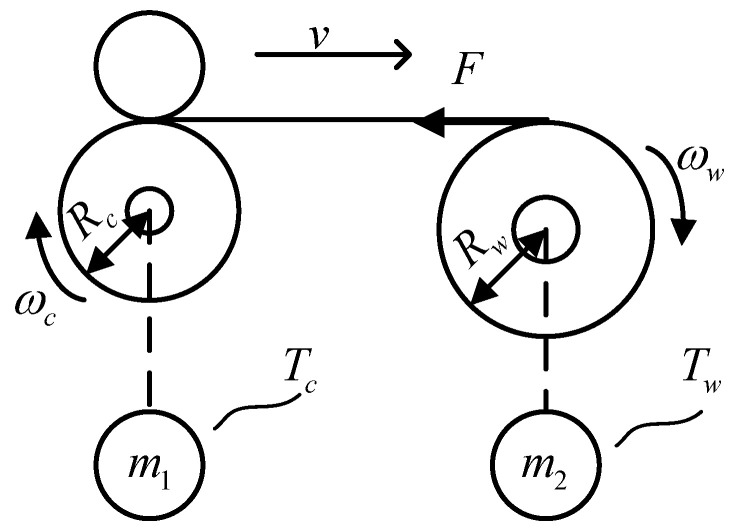
Diagram of the winding system structure.

The most basic unit of tension control is the two-motor tension system, which is a strongly coupled non-linear system, and the change in tension is associated with a variety of variables. The basic structure of the winding system is shown in Figure 2.

According to the principle of torque balance, the dynamic system equation is defined as

(13) $$\frac{d}{dt}\left(J_{w}\omega_{w}\right)\approx T_{w}-FR_{w}-T_{fw}-B\omega_{w}$$

where *J_w_* is the rotational inertia of the winding roller; *R_w_* is the radius of the winding roller; *ω_w_* is the angular velocity of the winding roller; *T_w_* is the electromagnetic torque of the winding motor; *T_fw_* is the coulomb friction; *R_c_* is the radius of the conveyor roller; *ω_c_* is the angular velocity of the conveyor roller; *T_c_* is the electromagnetic torque of the conveyor motor; and *B* is the coefficient of viscous friction. The winding motor is used to control the linear velocity of yarn movement, and the conveyor roller motor is employed to adjust the yarn tension.

Taking the tension, *F*, as a state variable, the tension generated during the same control cycle can be approximated as a constant value due to the high sampling frequency of the controller. The rate of change of the rotational inertia is much smaller than the rate of change of the angular velocity [29], and is defined as

(14) $$\left\{\begin{array}{l} \frac{d}{dt}\left(J_{w}\omega_{w}\right)\approx J_{w}\frac{d\omega_{w}}{dt}\approx T_{w}-F_{g}R_{w}-T_{fw}-B\omega_{w}\\ \frac{dF_{g}}{dt}=0 \end{array}\right.$$

where *F_g_* is the yarn tension observed by the tension observer. The equation for the state for the winding motor is generated from Equation (14) as

(15) $$\left\{\begin{array}{l} \dot{x}=Ax+Bu\\ y=Cx \end{array}\right.$$

where

$$\begin{array}{c} A=\left[\begin{array}{cc} -\frac{B_{w}}{J_{w}} & -\frac{R_{w}}{J_{w}}\\ 0\; & 0 \end{array}\right]\;\;\;B=\left[\begin{array}{c} \frac{1}{J_{r}}\\ 0 \end{array}\right]\;\;\;C=\left[1\;0\right]\\ x={\left[\begin{array}{cc} \omega_{w} & F_{g} \end{array}\right]}^{T}\;\;\;y=[\omega_{w}]\;\;\;u=[T_{w}-T_{fw}] \end{array}$$

Using the angular velocity of the winding roller as a correction quantity for the observer, the tension observer is constructed as

(16) $$\left\{\begin{array}{l} \dot{\hat{x}}=A\hat{x}+Bu+K\left(y-\hat{y}\right)\\ \hat{y}=C\hat{x} \end{array}\right.$$

where: $\hat{x}$ is the observed state variable; $\hat{y}$ is the output of the observer; and ***K*** is the feedback gain.

$$\hat{x}={\left[\begin{array}{cc} {\hat{\omega }}_{w} & {\hat{F}}_{g} \end{array}\right]}^{T}\;\;\;\hat{y}=[{\hat{\omega }}_{w}]\;\;\;K={\left[k_{1}k_{2}\right]}^{Τ}$$

In order to obtain a fast, noise-resistant and relatively stable tension-observation system, a suitable feedback matrix, which is mainly based on the pole configuration, is required. The poles are first located on the left half of the s-plane to make the system stable, and then the poles are chosen to balance the response speed and noise immunity of the observer. Assuming that the eigenvalues of the desired observer are *α* and *β*, respectively, we obtain

(17) $$\left\{\begin{array}{l} k_{1}=-\left(\alpha +\beta \right)-\frac{B_{w}}{J_{w}}\\ k_{2}=-\alpha \beta \frac{J_{w}}{R_{w}} \end{array}\right.$$

**Figure 3:**
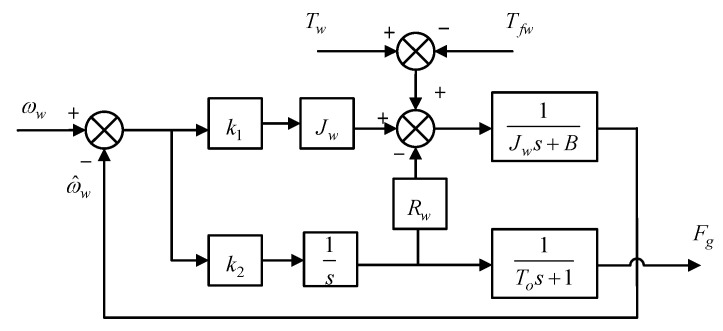
Diagram of the tension observer principle block.

The obtained yarn tension is then added to a first-order low-pass filter, *L*(*s*), to filter out external high-frequency noise disturbances such as motor vibrations. The filter is shown in Equation (18).

(18) $$L\left(s\right)=\frac{1}{T_{o}s+1}$$

where *T_o_* is the time constant of the low-pass filter. Different values of *T_o_* are chosen to correspond to the different filter cut-off frequencies [30]. A block diagram of the tension observer structure is shown in Figure 3.

The tension observer based on the motor-torque balance principle completes the estimation of the yarn tension using the motor electromagnetic torque and angular velocity, without taking into account the effect of the actual yarn oscillation on the observed tension.

### 2.3. Adaptive Weighted Fusion Model

**Figure 4:**
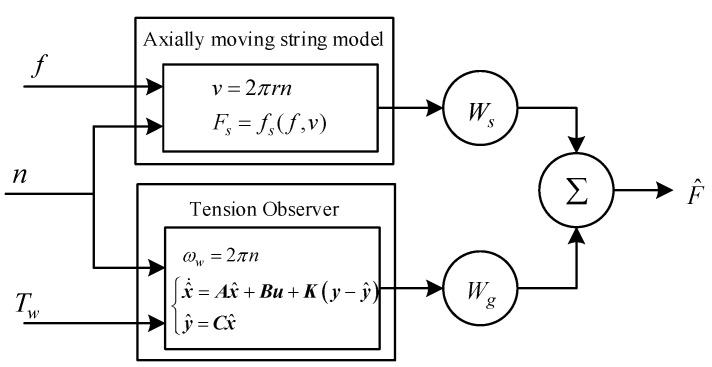
Adaptive weighted fusion algorithm structure.

Two mathematical models of indirect tension estimation sometimes do not take some variables into account. Therefore, the estimated tension values need to be fused with each other to improve measurement accuracy and fault tolerance. The adaptive weighted fusion algorithm is easy to implement in the PLC as the controller in most textile sites. The weight coefficient of each sensor can be adjusted in real-time based on changes in the variance of the tension values, such that the mean square error of the fusion system is kept to a minimum and optimal fusion effect is achieved [31]. The fusion model is shown in Figure 4.

In Figure 4, *n* is the rotational speed of the motor. $\hat{F}$ is the tension value after data fusion. The result of the tension fusion is written as

(19) $$\hat{F}=W_{s}F_{s}+W_{g}F_{g}$$

where *W* is the weight coefficient for each method, and

(20) $$W_{s}+W_{g}=1$$

Based on the theory of multivariate functions for extreme values, the total mean square error is minimized when the weight coefficient is as presented in Equation (21).

(21) $$W_{s}=\frac{1}{\sigma_{s}{}^{2}\left(\frac{1}{\sigma_{s}{}^{2}}+\frac{1}{\sigma_{g}{}^{2}}\right)}$$

The data detected by each method are assumed to be *X*_1_, *X*_2_, …, *X_n_* and the population variance is written as

(22) $$\sigma_{s}{}^{2}=\frac{1}{n}\left[{\sum_{k=1}^{n}\left(X_{k}-\bar{X}\right)}^{2}\right]\;\;\;\;\left(k=1,2,\ldots ,n\right)$$

Similarly, $\sigma_{g}{}^{2}$ can be obtained and the mean value is written as

(23) $$\bar{X}=\frac{1}{n}\sum_{k=1}^{n}X_{k}$$

*W_g_* can be obtained as shown above. The weight coefficient changes as the variance in the tension values obtained using two detection methods change and current measurements are combined to achieve optimal fusion effect. Since the tension value observed using the tension observer is a supplement to the tension value estimated using machine vision, the initial value is more dependent on the tension value detected by the machine vision. Therefore, the initial value of *W_s_* is determined as 1 and the initial value of *W_g_* is determined as 0. The overall design concept of a dynamic yarn tension detection system is established by combining the vibration model with a tension observer. The system is divided into three parts: axially moving string model to estimate tension, tension observer to observe tension and adaptive-weighted tension fusion. The yarn tension detected by machine vision is combined with the tension obtained by the tension observer to achieve the most stable, real-time results possible.

## 3. Machine Vision Tension Detection

### 3.1. Image Acquisition

**Figure 5:**
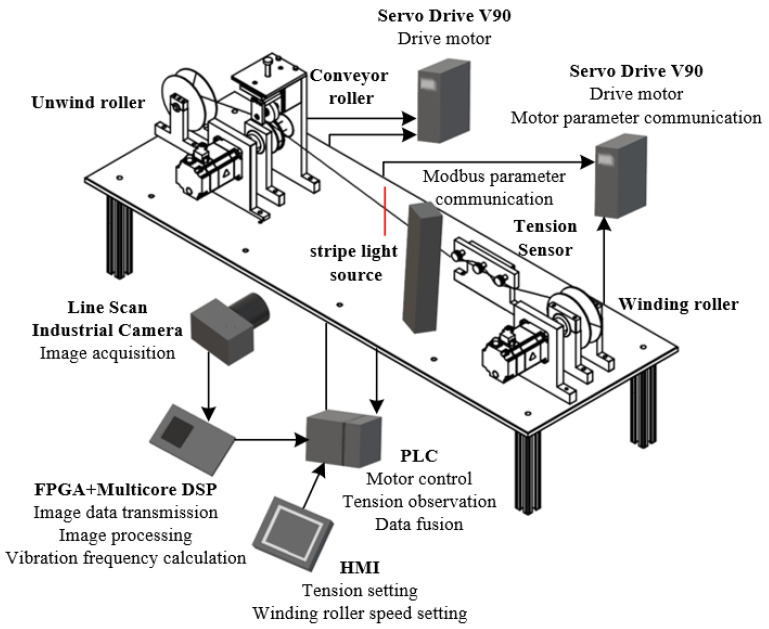
Mechanical structure of the detection system.

The winding roller motor runs and the yarn travels at a set velocity through the conveyor rollers. A line scan camera is used to quickly obtain images of the moving yarn. It is installed at a suitable location to obtain images of the yarn at a point of lateral vibration, as shown in Figure 5.

The objective is to capture information on the vibrating yarn image. A large field depth is not required to highlight the subject, but a suitable field of view needs to be selected in order to obtain sufficient yarn vibration. To make the yarn outline clear and reduce interference, the stripe light source—with adjustable brightness—is enabled. After selecting the appropriate object distance, the depth of the field and the field of view are obtained by adjusting the focal length and aperture. The formula for calculating the field of view is

(24) $$\frac{\mu N}{f_{o}}=\frac{l_{o}}{d}$$

where *μ* is the image element size; *N* is the number of pixels occupied by the object image in a single direction; *f_o_* is the camera focal length; *l_o_* is the actual field of view in the direction of vibration; and *d* is the object distance [32]. Based on Equation (24), the camera is mounted at 430 mm, the same level as the yarn, and the line frequency of the line scan camera is set to 10 kHz. The flexible parallel structure of the FPGA is used as the motion yarn-image acquisition unit and pre-processing unit. DSP and FPGA are connected to each other using the SRIO protocol to achieve high-speed connection of the heterogeneous processors. The FPGA quickly transmits the collected and processed yarn greyscale image data to the DSP, resulting in real-time signal processing.

### 3.2. Yarn Image Processing

The images captured by the line scan camera can be seen as discrete time series. Each frame represents a different point in time. Since the line scan camera is set to capture images at a line frequency of 10 kHz, the time for each image line is determined to be 0.1 ms. To have a suitable range of tension measurements, a set of yarn vibration peaks and troughs needs to be obtained. The line scan camera is chosen to give a tension solution at every full 512 × 512 resolution capture, which is also a sampling period of 0.0512 s. Acquisition of the yarn images and image processing occur simultaneously, i.e., the DSP performs image processing while the line scan industrial camera is still capturing images. This does not affect the speed of detection but requires image processing to occur at a faster rate than image acquisition.

#### 3.2.1. Overall Image Processing Process

**Figure 6:**
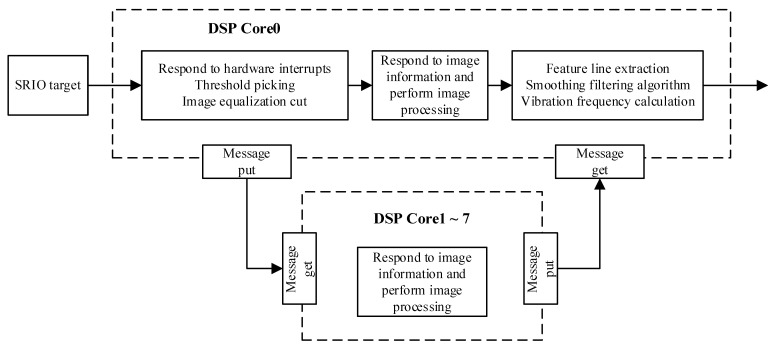
DSP multi-core image processing flow chart.

Yarn image processing is based on a multi-core DSP processor with embedded real-time cores and parallel image processing, making it capable of meeting the performance requirements of real-time data processing in many industrial fields. The overall flow of yarn image processing is shown in Figure 6.

TMS320C6678 is a high-performance floating point embedded 8-core digital signal processor. Yarn greyscale image data from the FPGA is received by the multi-core DSP processor as a Serial Rapid I/O (SRIO) target. The main core, Core0, responds to hardware interruption, selects a threshold for image segmentation and divides the image equally. The segmented yarn image data is subsequently sent to the DSP’s slave cores via the Inter-Process Communication Message Queue (IPC MQ). The slave cores simultaneously respond to image information and process the images, which reduces the time required to run the single-core-only program. The feature lines of the vibrating yarn are extracted and smoothed based on the processed image data. Finally, the vibration frequency is calculated. The overall design idea for the program is to minimize the running time of the program on the premise that yarn vibration frequency can be calculated.

#### 3.2.2. Image Segmentation Threshold Selection

**Figure 7:**
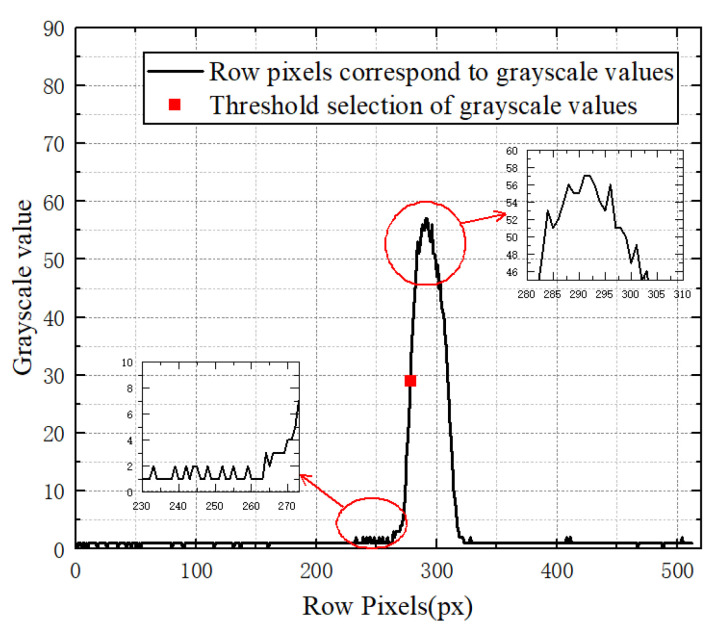
Yarn image row pixel greyscale values.

The regions of interest are obtained conventionally from banded images by scanning the image line by line and determining the upper and lower boundary points or the brightest point [33]. This method increases the algorithm running time as the image resolution increases and the region of interest decreases. The greyscale values of row pixels of the yarn image are shown in Figure 7.

If the upper and lower boundary points or the brightest point are directly selected as thresholds during image processing, the results will be unfavourable for the subsequent calculation of vibration frequency. The more stable the grey value is, the closer it is to the centre point of the rise or fall, thus the brightest point’s grey value, as the reference, is divided in half and used as the threshold value for image segmentation. This method only needs to identify the threshold value of the first image row, hence not only excludes the uncertainty in finding the boundary point or the brightest point’s grayscale value, but also reduces the time required to find the brightest point in each row in cases where there are a lot of operations.

**Figure 8:**
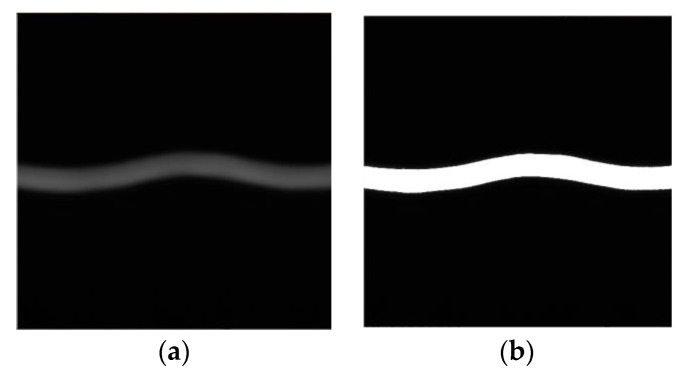
Yarn image processing effect. (**a**) Original image. (**b**) Threshold image segmentation.

The program is implemented in the DSP, thus the image processing results are viewed with the help of the YUV Player software. The image data is exported directly from the address memory by setting a breakpoint in the program. The image processing results are shown in Figure 8.

**Figure 9:**
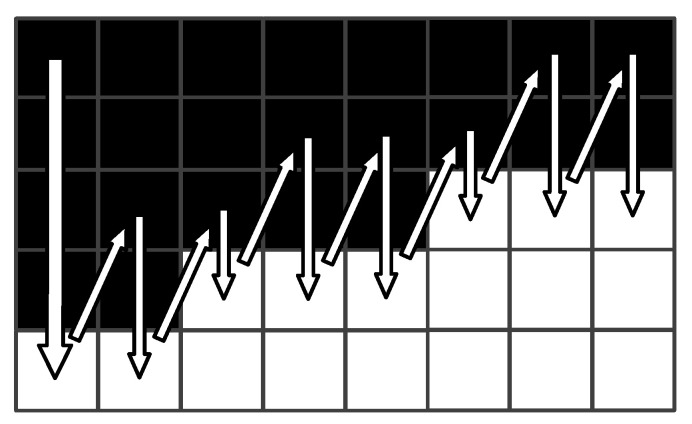
Principle diagram of feature-line fast-scanning algorithm.

The feature-line fast-scanning algorithm is designed to avoid wasting a lot of time scanning from the beginning of each row. First, the position with a grey value of 255 pixels is determined by searching for the first line of the yarn image. The starting value for the subsequent line scans is then decided by floating a fixed value based on the position of the previous moment. And the next boundary will be searched as long as find the brightest grey value. Ultimately, the data that characterise the information in the moving yarn image are obtained. The schematic of the algorithm is shown in Figure 9.

#### 3.2.3. One-Way Extreme Value Search Algorithm

**Figure 10:**
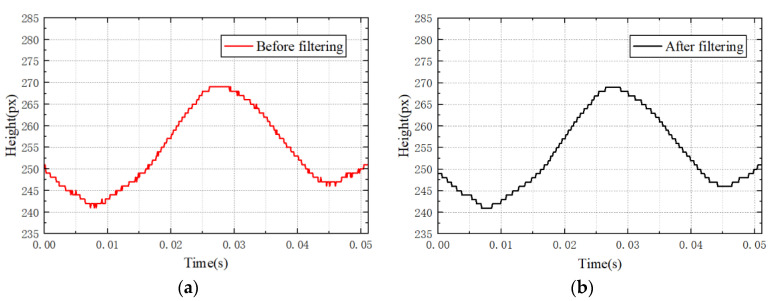
Yarn feature line results. (**a**) Before filtering. (**b**) After filtering.

Since the uneven density of the transversely vibrating yarns and micro-angle changes during light source irradiation, the signal noises are formed and the change-point detection results of the yarn image are affected by the noises [34]. The directly extracted feature line is shown in Figure 10a. The figure shows deviations in some points of the extracted feature line. In order to facilitate the determination of wave peaks and valleys and reduce errors, a smoothing filtering algorithm is used to filter out signal noise interference.

**Figure 11:**
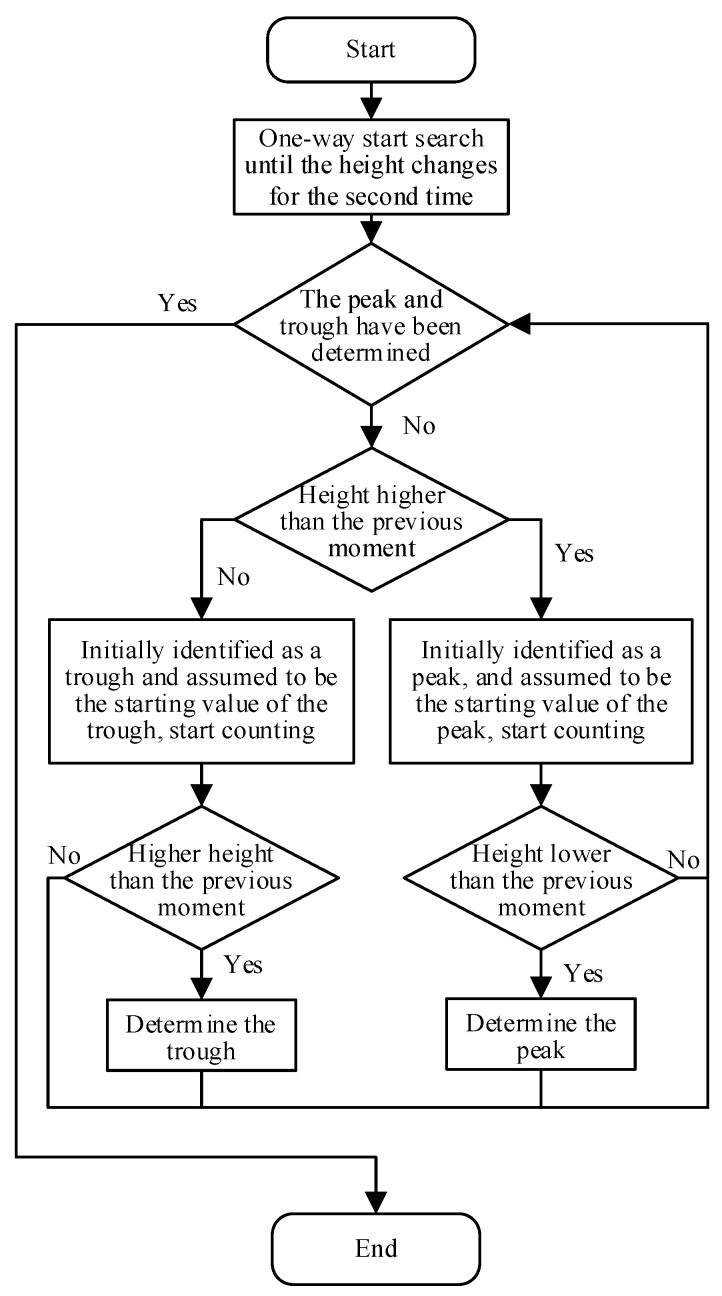
One-way extreme-value search algorithm.

The filtered feature line in Figure 10b shows that the extreme values cannot be taken directly as peaks and troughs. Therefore, the height of the yarn vibration is gradually scanned at each moment, and the moments in the middle of the poles are used as the peaks or troughs. To avoid directly determining the position of the starting part as a peak or valley, determination of the peak and valley positions commences when the scanning height changes. Before searching for the poles, it is necessary to confirm whether the peaks and valleys have been determined. When the current height in the wave peak determination process is higher than that of the previous moment, it is initially determined as the peak, and the count is started. If the height changes again to a lower value, the position of the peak is determined based on the count value. Otherwise, the count is cleared and the peak needs to be determined again. The trough is determined in a similar manner. A one-way extreme-value scanning algorithm is designed as shown in Figure 11.

The yarn vibration frequency is calculated directly in half a cycle after scanning the first set of peaks and troughs, based on the line frequency set by the line scan camera, which communicates with the PLC through the serial port. If the estimated frequency exceeds the measurable range, the value obtained is judged to be incorrect and immediately abandoned. Finally, the yarn tension is estimated according to the approximate fitting formula.

## 4. Tension-Observer Tension Observation and Data Fusion

**Figure 12:**
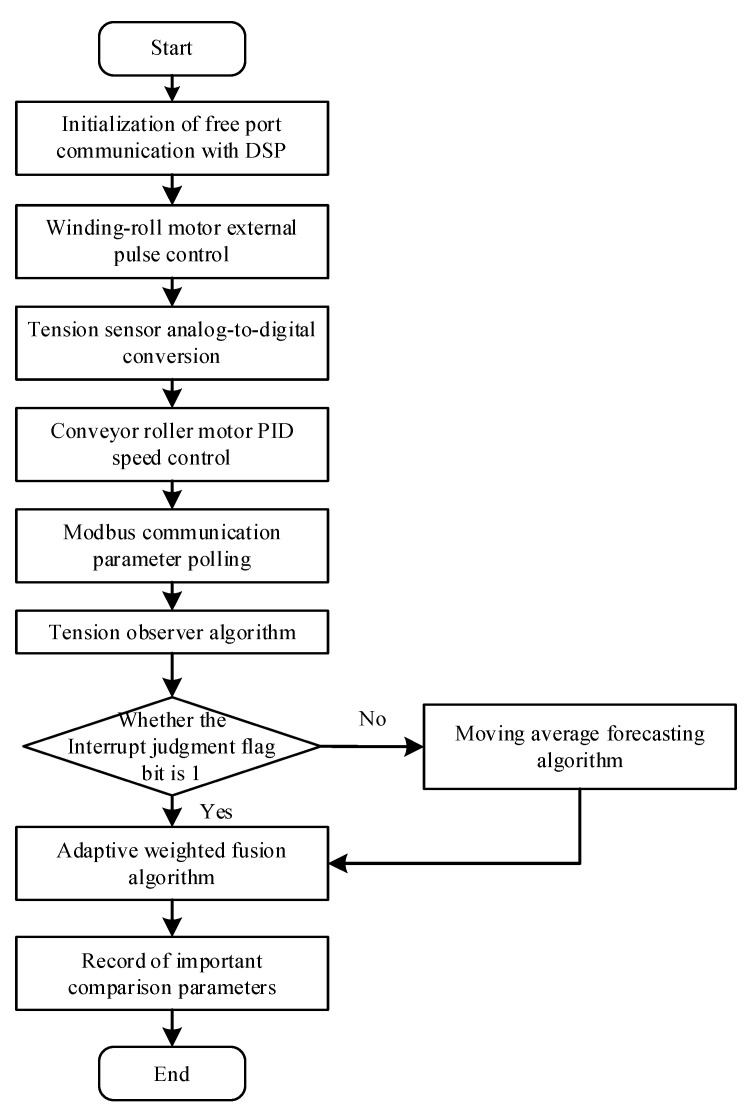
Flowchart of the PLC internal program.

The rotational inertia of the conveyor-roller motor is not easy to determine, thus a coordinated control system of the winding tension and speed is constructed with the winding-roller motor as the host and the conveyor-roller motor as the slave. The winding-roller motor is controlled by the high-speed pulse signal from the PLC to the servo drive; at the same time, the conveyor-roller motor works in a speed-control mode, where the maximum speed is constrained by the host speed [35]. Through Modbus polling communication, the electromagnetic torque and angular velocity information of the motor are obtained from the servo drive in real time. The tension observer algorithm is then run inside the controller to facilitate observation of the yarn tension. The internal PLC program implementation is shown in Figure 12.

During algorithm fusion, the insufficient sampling rate of machine vision detection makes it difficult to achieve effective adaptive weighted fusion with the tension observer’s observed tension, hence a moving average prediction algorithm is employed to infer missing data due to the machine vision sampling rate based on historical data [36]. A flag bit is set to determine whether the yarn vibration frequency calculated by the machine vision section is transmitted to the PLC to switch the tension value.

The adaptive weighted fusion algorithm improves the measurement accuracy of the multi-sensor data fusion by combining the tension detected using machine vision with the tension data observed using the tension observer; however, the effect of the delay in measured tension values, from acquisition to calculation, also needs to be considered in order to achieve the best fusion results.

## 5. Experimental Test Platform

**Figure 13:**
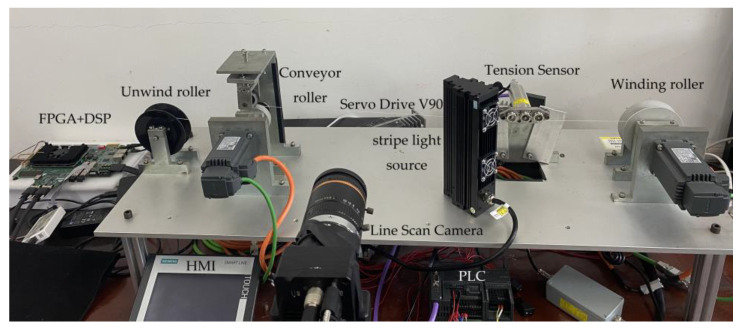
Construction of the experimental platform.

An experimental laboratory platform was created to evaluate the performance of the proposed detection system. The system can be divided into three parts: machine vision detection, tension-observer observation and yarn tension control. The implementation of the tension observer is correlated with the control part, which mainly includes a human–machine interface (HMI), programmable logic controller (PLC), servo drive and servo motor, to realise the tension observer and coordinated control of the tension and speed in the spinning process of the fabric-based coils. The machine vision part includes a line scan industrial camera, FPGA and multi-core DSP to complete tension detection based on the yarn vibration image data. A simple winding-control system is built by simulating the spinning process. The yarn-tension detection platform is shown in Figure 13.

The experimental system includes the control part of the HMI device (Smart 700 IE V3), a Siemens PLC (S7-200smart) with two Siemens servo drives and motors and winding materials. A line scan camera (Hikvision MV-CL042-91CM), a stripe light source and a lens (MVL-LF5040M-F) are selected for image acquisition. The image processing part constitutes an FPGA chip (Xilinx Kintex-7) and a floating-point DSP chip (TMS320C6678). In addition, the tension sensor (ZHZL-HL), which has an accuracy of 0.5%, is installed in the experimental platform for comparison. The specific parameters of the experimental system are: line scan industrial camera resolution, 512 × 2; line frequency, 10 kHz; lens aperture, F4.0; servo motor power, *p* = 0.4 kW; rated speed, *n* = 3000 r/min; rotational inertia, *J* = 3.51 × 10^−5^ kg·m^2^; torque, 1.27 Nm, where the winding material is Kevlar; yarn linear density, 230.769 tex; and yarn diameter, 0.6 mm.

## 6. Presentation and Discussion of Test Results

To verify the accuracy of the system’s results, the yarn tension measured by the contact-tension sensor was compared with the tension detected by the non-contact machine vision, the tension observed by the tension observer and the final achieved tension after data fusion. The experiments were carried out at an ambient temperature of 25 °C and a relative humidity of 40%.

**Figure 14:**
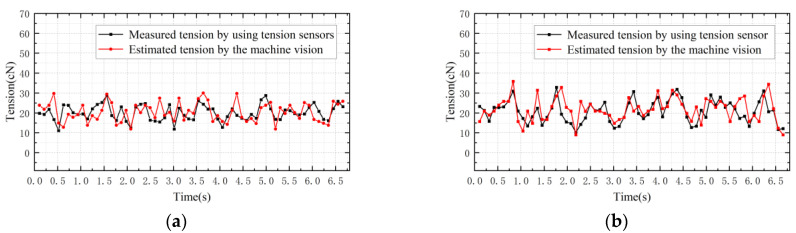
The sampling period is 0.104 s. (**a**) Motor speed is 100 r/min. (**b**) Motor speed is 150 r/min.

Figure 14 shows comparisons between estimated tension based on the machine vision and measured tension using the tension sensor when the tension sampling period is 0.104 s, the pre-set tension is 20 cN and the winding-roll motor is set to different speeds. The relative error between the tension estimated using machine vision and the tension measured using a contact sensor is shown in Table 1. It can be seen that estimated tension based on machine vision fluctuates around the pre-set tension over time and agrees with the tension obtained using the contact tension detector at approximately 10 tension values per second of output; the relative error is generally less than 14%. At the same time, tension detection can also be achieved by changing the motor speed to 150 r/min, as shown in Figure 14b. Discrepancies in the tension detection results of the two methods may be due to the fact that the machine vision method is based on the vibration frequency of the yarn and does not take into account the disturbance formed on the yarn during the operation of the motor. In the theoretical model of machine vision tension detection, the yarn density is considered to be constant under ideal conditions, but the linear density may change due to repeated stretching of the yarn during the experiment, leading to errors. Measured tension values may also be affected by smooth filtering on raw yarn data in the image data processing.

**Figure 15:**
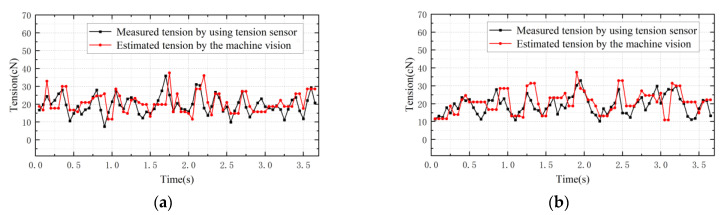
The sampling period is 0.05 s. (**a**) Motor speed is 100 r/min. (**b**) Motor speed is 150 r/min.

When the tension-detection system needs to output 20 tension values per second, a sampling period of 0.05 s is set. Figure 15 shows the comparisons between estimated tension based on the machine vision and the tension measured using tension sensor when the tension sampling period is 0.05 s and the pre-set tension is 20 cN. The tension of the yarn can still be detected when the sampling rate is faster and the motor speed is 100 r/min, as shown in Figure 15a. However, the processing speed provided by the vision solution within a single sampling time is not sufficient to meet the solution requirements. The tension values estimated using machine vision are often discontinuous, resulting in some tension values not being sampled on time, hence affecting the effectiveness of tension detection. The same procedure is repeated for the yarn when the motor speed is 150 r/min, and the result comparisons are presented in Figure 15b. The detection results are in agreement with the preceding conclusions.

**Figure 16:**
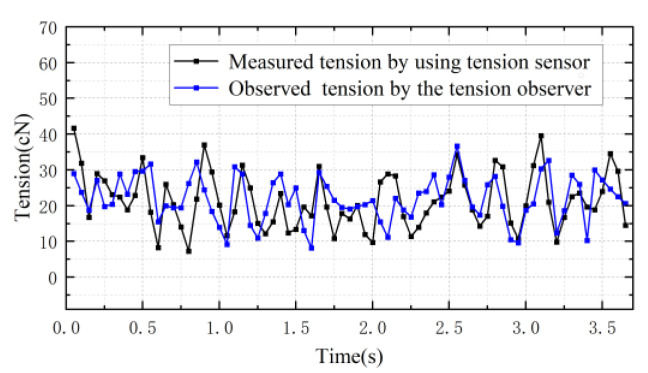
Comparisons between tension-observer tension observations.

The tension values observed using the tension observer and the tension values measured using the contact tension detector when the motor speed is 100 r/min, the pre-set tension is 20 cN and the tension sampling period is 0.05 s are shown in Figure 16. Table 2 presents the relative error between the tension observed using the tension observer and the tension measured using a contact sensor. The tension observer also fluctuates around the pre-set tension over time, and the sampling rate is not limited. However, the relative error is generally less than 17%, and may be due to the fact that the actual yarn oscillations are not taken into account during the continuous winding of the yarn. Moreover, it may also be impacted by the accuracy of the measurement of motor parameters and the variation in rotational inertia.

**Figure 17:**
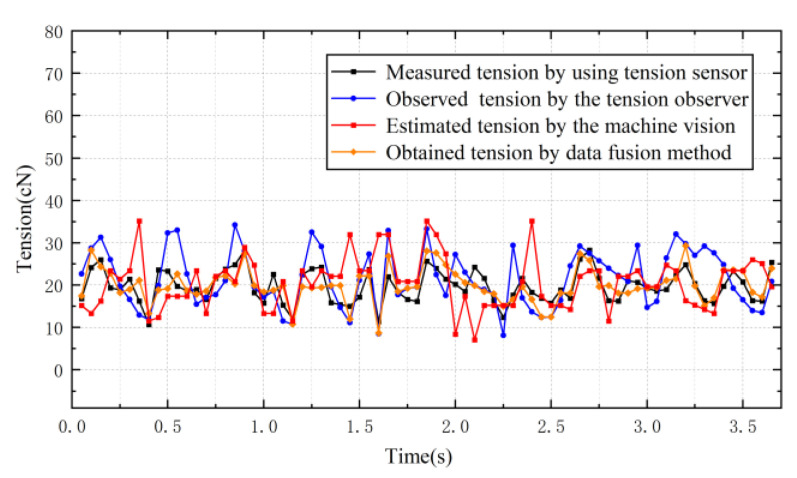
Experimental comparisons of adaptive-weighted tension fusion.

**Figure 18:**
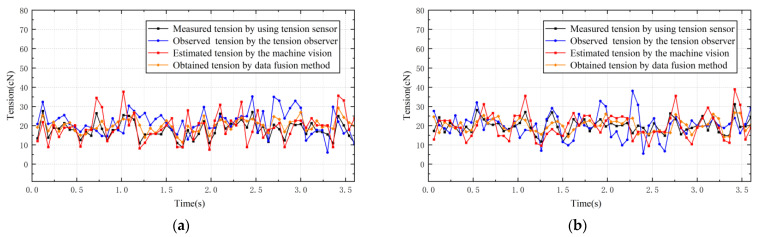
Data fusion experiments. (**a**) The sampling period is 0.06 s. (**b**) The sampling period is 0.07 s.

Figure 17 shows the comparison between estimated tension based on the machine vision, the tension values observed using the tension observer, the fusion tension and the tension measured using contact tension sensors when the tension sampling period is 0.05 s and the pre-set tension is 20 cN. Relative errors of data fusion tension detection are shown in Table 3. The combined tension values correlate with the tension values measured using the contact tension sensor, with the error generally within 10%. Under the condition that the sampling rate increases, the missing tension values generated using machine vision methods are predicted using the moving average prediction algorithm. The adaptive weighted fusion algorithm solves the sudden change situation where the two individual detection methods are affected and generate large errors, making the detected tension value relatively stable. The differences in the tension test results are mainly caused by the accuracy of measurement of motor parameters, the effect of rotational inertia change and the method used for smooth filtering raw yarn data during image data processing. In addition, the yarn density changes during the multiple experimental stretches. The additional friction in the tension sensor also has unavoidable effects on the tension calculation results. The same procedure is repeated with all conditions except sampling time kept constant, as shown in Figure 18. The comparisons between the results are basically consistent with those of the analysis above.

**Figure 19:**
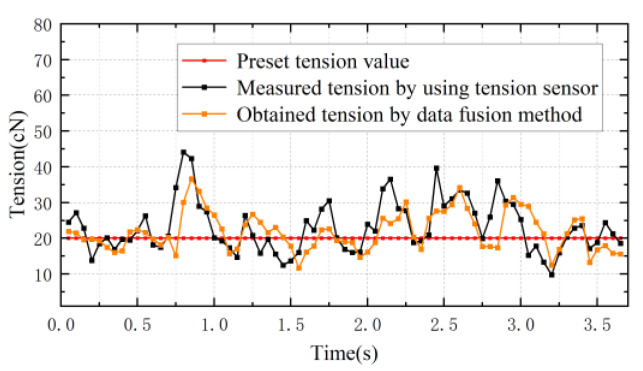
Tension control experiment using data fusion method.

Finally, the contact tension sensor is replaced with the tension value detected using data fusion to represent the feedback value of the controller to control the conveyor roller motor and check the yarn tension control effect when the motor speed is 100 r/min, the pre-set tension is 20 cN and the tension sampling period is 0.05 s, as shown in Figure 19. The yarn tension value basically fluctuates around the pre-set tension value over time, indicating that the designed detection system can be used for tension control. However, it can be seen that the yarn tension value deviates from the pre-set tension value within 2–3 s. More research is needed to improve the detection system.

## 7. Conclusions

In summary, a dynamic yarn-tension detection system was developed by combining machine vision with a tension observer. Machine vision is employed to capture and process the images of vibrating yarns, and the calculated yarn tension value is then combined with the value obtained from the tension observer, using an adaptive weighted data fusion method to improve the sampling rate and detection accuracy to achieve the non-contact measurement of dynamic yarns. The performance of the system was investigated using the simulated spinning experiment platform, which showed that the proposed method is effective. Some issues remain for further research, in particular the smaller range of the tension sensors with higher accuracy, the accuracy of measuring motor parameters, the effect of rotational inertia change, and the method for finding peaks and valleys, which should improve the system. At the same time, the actual application results should be highlighted in the spinning production site, and the error rate of the system should be verified and corrective measures proposed. With continuous improvements in the various motor control algorithms, hardware design and signal processing scheme, the accuracy of the system can be improved further, and is expected to be used in more industrial applications.

## Figures and Tables

**Table 1:** Relative error in machine vision detection.

Tension Measured Using Tension Sensor (cN)	Tension Estimated Using Machine Vision (cN)	Relative Error (%)
19.57	20.94	7.00
21.09	18.79	−10.91
19.71	20.12	2.08
19.84	21.55	8.62
19.65	22.30	13.49
19.00	20.30	6.84
21.13	19.62	−7.15
19.24	20.13	4.63
20.40	18.10	−11.28

**Table 2:** Relative error of tension-observer observations.

Tension Measured Using Tension Sensor (cN)	Tension Obtained Using the Observer (cN)	Relative Error (%)
27.43	23.95	−12.69
21.12	24.14	14.30
20.19	20.52	1.63
20.12	22.65	12.45
17.73	20.40	15.06
18.74	18.10	−3.42
18.87	22.07	16.90
23.89	26.13	9.38
24.07	20.30	−5.66
19.29	21.87	13.37

**Table 3:** Relative error of data fusion yarn tension detection.

Tension Measured Using Tension Sensor (cN)	Tension Obtained Using Data Fusion Method (cN)	Relative Error (%)
20.42	21.62	5.88
18.78	18.44	−1.81
22.12	21.24	−3.98
18.46	18.43	−0.16
17.74	18.51	4.34
21.39	23.34	9.12
17.85	16.68	−6.55
20.54	20.22	−1.56
19.58	19.55	−0.15
20.14	21.70	7.75

## Data Availability

The data presented in this study are available from the corresponding author (J.Y.) upon reasonable request.
